# A prospective cohort study of neighborhood stress and ischemic heart disease in Japan: a multilevel analysis using the JACC study data

**DOI:** 10.1186/1471-2458-11-398

**Published:** 2011-05-27

**Authors:** Yoshihisa Fujino, Naohito Tanabe, Kaori Honjo, Sadao Suzuki, Kokoro Shirai, Hiroyasu Iso, Akiko Tamakoshi

**Affiliations:** 1 Department of Preventive Medicine and Community Health, University of Occupational and Environmental Health, Kitakyushu, Japan; 2 Department of Health and Nutrition, Faculty of Human Life Studies, University of Niigata Prefecture, Niigata, Japan; 3 Osaka University Global Collaboration Center, Osaka, Japan; 4 Department of Public Health, Nagoya City University Graduate School of Medical Sciences, Aichi, Japan; 5 Department of Human Sciences, School of Law and Letters, University of the Ryukyus, Okinawa, Japan; 6 Department of Social and Environmental Medicine, Graduate School of Medicine, Osaka University, Osaka, Japan; 7 Department of Public Health, Aichi Medical University School of Medicine, Aichi, Japan

**Keywords:** Multilevel Analysis, Japan, Stress, Cohort Studies, Coronary Disease

## Abstract

**Background:**

A body of research has shown that neighborhood environment may have an effect on a variety of health outcomes, including cardiovascular disease. One explanation for the mechanism of the effect of neighborhood on cardiovascular disease is psychosocial pathways. Direct evidence for an effect of neighborhood on cardiovascular disease with adjustment for perceived stress at the individual level has not been obtained, however. The Japan Collaborative Cohort Study for the Evaluation of Cancer Risk provides a unique dataset which has aggregated area-based cohorts from 45 areas throughout Japan. The purpose of the present study was to examine the contextual effect of area-level stress on ischemic heart disease using data from a large prospective cohort in Japan.

**Methods:**

A baseline survey of 110,792 residents of 45 areas aged 40-79 years was conducted between 1988 and 1990. Analysis was restricted to subjects from the 33 of 45 areas providing information about self-rated stress (32183 men and 45896 women). Multilevel Poisson regression models were employed in a two-level structure of individuals nested within the 33 areas. Area-level stress was calculated by sex as the number of persons who rated their stress level as high divided by the total number of subjects in that area. Mortality rate ratios (MRRs) per 1 percentage point increase in area-level stress were estimated with adjustment for compositional individual factors.

**Results:**

During 15 years of follow-up (1,116,895 person-years), 936 deaths due to ischemic heart disease were recorded. Area-level stress varied from 6% to 22%. In the multivariable models, MRRs of area-level stress were 1.06 (95% confidence interval: 1.00-1.12, p = 0.043) in men and 1.07 (95% confidence interval: 1.00-1.14, p = 0.057) in women.

**Conclusions:**

Area-level stress affects the likelihood of death due to ischemic heart disease of individuals in men. The present findings may suggest that stress should be considered not only within the individual but also within the neighborhood context.

## Background

A growing body of research has indicated a possible effect of neighborhood and residential environment on a variety of health outcomes, including cardiovascular disease. In particular, neighborhood socioeconomic characteristics, such as deprivation, income inequality, and social network, have been associated with cardiovascular disease[1-8].

One plausible mechanism of the link between neighborhood environment and the development of cardiovascular disease is psychosocial pathways. It has been shown that neighborhood characteristics of a poor built environment are associated with psychosocial stress [9,10]. It is also possible that the biological pathway between neighborhood characteristics and cardiovascular disease is mediated by an abnormal neuroendocrine secretory pattern due to stress. Living in a stressful neighborhood may discourage residents from adopting important lifestyle measures such as physical activity, which in turn may lead to the development of hypertension [10].

Nevertheless, no direct evidence for a neighborhood effect on cardiovascular disease with adjustment by perceived stress at the individual level has yet been obtained, despite the fact that stress at the individual level is regarded as a risk for the development of ischemic heart disease [11-14]. Moreover, most previous studies have measured stress at the neighborhood level indirectly by crime, violence, and the physical built environment.

One way to clarify the contextual effect of a stressor using data nested by area is with a multilevel analysis [15-17]. The Japan Collaborative Cohort Study for the Evaluation of Cancer Risk (JACC Study) provides a unique dataset which aggregates area-based cohorts from 45 areas throughout Japan [18-21].

Here, we examined the contextual effect of area-level stress on mortality due to ischemic heart disease using data from this large prospective cohort in Japan.

## Methods

**Figure 1:**
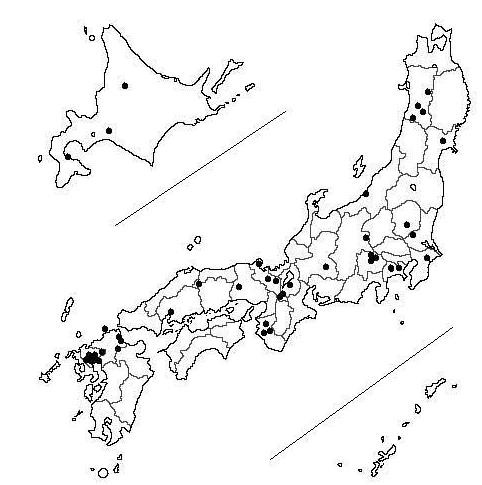
**Locations of the study areas in Japan**.

The JACC Study was sponsored by the Ministry of Education, Science, Sports and Culture of Japan and has been described in detail elsewhere [18-21]. Briefly, a baseline survey was conducted in 45 areas of Japan from 1988 until 1990 among 110,792 residents (46,465 men and 64,327 women) who ranged in age from 40-79 years at recruitment. The areas consisted of three towns in Hokkaido; five towns in the Tohoku region; five towns in the Kanto region; one city, three towns and two villages in the Chubu region; eight towns and two villages in the Kinki region; one city and one town in the Chugoku region; and four cities, nine towns and one village in Kyushu (Figure 1). In 22 of 45 areas, all residents living in the given target area were regarded as study subjects. In the other areas, the study subjects consisted of health checkup examinees plus volunteers. Response rates were approximately 83% [20]. The vital status of each participant was followed until the end of 2006 using data held at regional health centers, with permission from the Ministry of Public Management, Home Affairs, Post and Telecommunications of Japan to review population registration entries and death certificates.

The JACC study was approved by the Ethics committee of Nagoya University, because one of the authors, Dr. Akiko Tamakoshi who is a current principal investigator formerly belonged to Nagoya University.

### Data retrieval for analysis

Of the 45 areas, 12 used a slightly different questionnaire which had no questions on perceived mental stress. Analysis was therefore restricted to subjects from the 33 of 45 areas which obtained information about perceived mental stress (32,183 men and 45,896 women).

### Exposure Data

We used the participant's response to a question regarding stress which asked "What is the level of stress in your daily life?", with the four possible answers of extremely high, high, medium, or low. Area-level stress was calculated as the number of persons who rated their stress level as extremely high divided by the total number of subjects in that area.

The self-administered questionnaire also inquired about other baseline characteristics that were potentially related to mortality and ischemic heart diseases, including individual-level stress; smoking status (never, former or current smoker); alcohol intake (non-habitual drinker, former habitual drinker; or habitual drinker); history of cerebrovascular disease, hypertension, myocardial infarction, cancer, or diabetes; and hours of walking per day (< 0.5, 0.5, 0.6 to 0.9, and ≥ 1.0 hours per day).

### Statistical analysis

Mortality rate ratios (MRRs) per one percentage point increase in area-level stress were estimated using a two-level structure of individuals nested within the 33 areas estimated by multilevel Poisson regression models [17,22]. Two-level random-intercept and fixed slopes models were applied; these procedures take account of independence violation among individuals in the same cluster and eliminate the possibility that the ordinary least squares estimator might underestimate the true standard error [15-17].

Examining the contextual effects of community-level stress on individual health requires the adjustment of compositional individual factors. We used a multivariable model which added individual-level stress and other individual factors that are potentially associated with ischemic heart disease, including past medical history, smoking status, drinking behavior, and hours of walking per day. We also adjusted for type of recruitment (population-based versus health examinees and/or volunteers). All calculations were performed using STATA release 11 [23].

## Results

Additional file Table 1 shows the basic characteristics of study subjects and areas. During 15 years of follow-up (total 1,116,895 person-years; 451,897 person-years for men, and 664,997 person-years for women), 936 deaths due to ischemic heart disease were observed, with 546 deaths in men and 390 in women. Area-level stress varied from 6% to 22%.

Additional file Table 2 shows the results of the multilevel Poisson regression models for mortality due to ischemic heart disease in men. In the univariate model, area-level stress was not significant when random variation among areas was taken into account. However, after adjustment for compositional individual factors, including individual stress, area-level stress was significant. In the multivariable model, the MRR of area-level stress was 1.06 per 1 percentage point (95% confidence interval:1.00 - 1.12, p = 0.043). A significant association was also seen for stress at the individual level. Subjects who rated their stress as none showed a significant decrease in MRR (MRR = 0.79, 95% confidence interval: 0.62 - 0.99, p = 0.043).

Additional file Table 3 shows the results of the multilevel Poisson regression models for ischemic heart disease in women. The results were similar to those in men, although the multivariable models showed a marginally significant decrease in the MRR of area-level stress. In the multivariable model, the MRR of area-level stress was 1.07 per 1 percentage point (95% confidence interval: 1.00 - 1.14, p = 0.057).

## Discussion

The present study examined the presence of contextual effects of area-level stress on mortality due to ischemic heart disease in Japan. After adjustment of individual-level covariates, including stress at the individual level, area-level stress was significantly associated with a higher likelihood of death from ischemic heart disease in men. The borderline statistical significance observed in women could be related to fewer deaths.

The mechanisms by which area-level stress affects health are not clear, but several hypothetical explanations might be considered. First, the hypothetical term "area-level stress" is regarded as a reflection of social and environmental conditions. Social and environmental conditions may contribute to and result in stress at both the area and individual level. Area-level stress does not simply represent the average of aggregated individual characteristics, nor is it a proxy of individual characteristics, because stress derives from complex social structures and the location of individuals within them [24], and an individual's exposure to stress may be determined by residential context, to some extent at least [25]. Several studies have reported that perceived mental stress increases the risk of cardiovascular diseases [11-14]. However, given the physiological emphasis on stress and neuroendocrine relationships, these previous studies dealt with stress as a characteristic of individuals. Namely, they considered perceived individual mental stress as a risk factor that affected the individual subject only [11-14]; as the present findings indicate, however, the effect of area-level stress differs from that of individual stress, because an overall community stress can affect individuals.

Second, area-level stress may exert an effect on health via an effect on area-level characteristics. For example, people living in a higher area-level stress may have less capacity to build social capital within the area, which has recently come to be considered an important health determinant[26-28]. Matheson [10] also suggested that the stress created by neighborhoods impacts not only individual health, but may hamper the capacity of the population within that neighborhood to resist the pathological effects of ambient stress.

Third, area-level stress might measure potential neighborhood characteristics which might affect people's health. Several studies have shown that neighborhood characteristics measured by the physical built environment, income inequality, residential instability, social capital, violent crime, and ethnic diversity affect the individual's health, including cardiovascular diseases [1,5-8]. Many of these studies have argued that a plausible explanation for the association between neighborhood characteristics and cardiovascular disease is psychosocial stress, and a growing body of literature shows that chronic stressors in the local residential environment are associated with mental health [9,10,29-32]. Among those studies of neighborhood characteristics and cardiovascular disease, however, we are unaware of any which have considered perceived mental stress at the individual level. In the present study, area-level stress significantly affected the likelihood of death due to ischemic heart disease even after adjustment for perceived stress at the individual level. If the term "area-level stress" is in fact a proxy of neighborhood characteristics, it may actually reflect those neighborhood characteristics which affect cardiovascular diseases through a "materialistic pathway" rather than psychosocial pathways. The influence of "material conditions" on health often refers to the quality, type, and location of housing, food, transportation, and medical care; opportunities for cultural, recreational, and physical activities; and exposure to an array of environmental toxins [33].

Several limitations of the present study warrant mention. First, although we adjusted for selected individual factors that might potentially be associated with mortality, residual confounding was likely present at both the individual and area levels. A previous multilevel analysis using a representative sample of the general Japanese population reported that mortality from stroke was higher in rural than in urban areas [34]. Fukuda [35] also reported that mortality rates were associated with a wide range of socioeconomic conditions, including unemployment, old housing, primary health resources and density, education, public library activity, health check-up participation, and population growth. The impact of residual confounding by socioeconomic factors at the area level on the present results is uncertain. Although the present study showed a contextual effect of area-level stress on health, it is necessary to examine what area- and individual-level factors contribute to the association. Second, variables were assessed using simple self-administered questionnaires, but the validity and reliability of this methodology were not evaluated. In addition, variables assessed at baseline may have changed during follow-up. This estimation of effects based on baseline rather than time-varying data, which were not available, might have resulted in the attenuation of effects.

## Conclusions

In conclusion, this study showed that area-level stress affects the likelihood of death due to ischemic heart disease at the individual level in men. This effect remained after adjustment for perceived mental stress at the individual level. The present findings may suggest that stress should be considered not only within an individual but also within a neighborhood context.

## Competing interests

The authors declare that they have no competing interests.

## Authors' contributions

YF conceived the study, carried out the main analyses, and wrote the manuscript. NT participated in the design of the study and performed the statistical analysis. KH participated in the design and coordination of the study and helped draft the manuscript. SS participated in the design and coordination of the study and helped draft the manuscript. KS participated in the design and coordination of the study and helped draft the manuscript. HI participated in the design and coordination of the study and helped to draft the manuscript. TA participated in the design and coordination of the study and helped draft the manuscript. All authors have read and approved the final manuscript.

## Pre-publication history

The pre-publication history for this paper can be accessed here:

http://www.biomedcentral.com/1471-2458/11/398/prepub

## Supplementary Material

Additional file 1 Table 1: Basic characteristics of study subjects and areas Click here for file

Additional file 2 Table 2: Mortality rate ratios (MRR) for ischemic heart disease in men Click here for file

Additional file 3 Table 3: Mortality rate ratios (MRR) for ischemic heart disease in women Click here for file
