# Supplementary material for: A prospective cohort study of neighborhood stress and ischemic heart disease in Japan: a multilevel analysis using the JACC study data
Source: BMC Public Health. 2011 May 27;11:398. doi: 10.1186/1471-2458-11-398 (PMC3128019; doi:10.1186/1471-2458-11-398)
Supplement: Additional file 1 — Table 1: Basic characteristics of study subjects and areas [file 1471-2458-11-398-S1.PDF]

Table 1 Basic characteristics of study subjects and areas

Variable	Men n=32183	Women n=45896
No. of deaths	546	390
Person-years	451897	664997
Individual variables		
Mean age (SD)	57.5 (10.2)	57.7 (10.0)
Stress		
High	11.2	10.7
Moderate	11.6	9.4
Low	60.3	62.6
None	16.9	17.3
Past medical history (%)		
Cerebrovascular disease	2.1	1.0
Hypertension	19.9	21.4
Myocardial infarction	2.9	2.8
Diabetes	6.5	3.9
Cancer	1.0	1.7
Smoking status (%)		
Current smoker	50.6	4.7
Former smoker	25.4	1.4
Never smoker	19.7	82.0
Missing	4.3	11.9
Alcohol intake (%)		
Habitual drinker	71.9	22.0
Former habitual drinker	6.4	1.8
Non-habitual drinker	17.6	69.1
Missing	4.1	7.2
Walking hours per day (%)		
≥ 1.0	45.5	46.6
0.6-0.9	18.4	19.2
0.5	17.3	15.9
< 0.5	10.2	8.3
Missing	8.6	10.0
Area-level variable (n=33)		
Range of area-level stress (%)*	6% - 22%	
Mean of area-level stress (SD)	9.6 (0.5)	

* showing both sexes.
