# Supplementary material for: A prospective cohort study of neighborhood stress and ischemic heart disease in Japan: a multilevel analysis using the JACC study data
Source: BMC Public Health. 2011 May 27;11:398. doi: 10.1186/1471-2458-11-398 (PMC3128019; doi:10.1186/1471-2458-11-398)
Supplement: Additional file 2 — Table 2: Mortality rate ratios (MRR) for ischemic heart disease in men [file 1471-2458-11-398-S2.PDF]

Table 2. Mortality rate ratios (MRR) for ischemic heart disease in men.

	Person-year	No. of death	Univariable				Multivariable			
			MRR	95%CI		p	MRR	95%CI		p
Area-level stress (per 1 percentage point)			1.05	0.99	1.12	0.119	1.06	1.00	1.12	0.043
Age (per 1 year)	451897	546	1.10	1.09	1.11	<0.001	1.09	1.08	1.10	<0.001
Stress										
High	53000	45	0.60	0.44	0.83	0.002	0.92	0.67	1.26	0.603
Moderate	52791	45	0.63	0.46	0.86	0.003	0.91	0.67	1.25	0.573
Low	271699	360	Reference				Reference			
None	74408	96	0.90	0.71	1.13	0.365	0.79	0.62	0.99	0.043
History of stroke (no)	445115	524	Reference							
History of stroke (yes)	6783	22	2.73	1.78	4.20	<0.001	1.21	0.78	1.88	0.393
History of hypertension (no)	370203	344	Reference							
History of hypertension (yes)	81695	202	2.64	2.21	3.14	<0.001	1.74	1.45	2.09	<0.001
History of ischemic heart disease (no)	441733	484	Reference							
History of ischemic heart disease (yes)	10164	62	5.39	4.10	7.08	<0.001	2.99	2.26	3.94	<0.001
History of diabetes (no)	426496	488	Reference							
History of diabetes (yes)	25401	58	1.92	1.46	2.53	<0.001	1.36	1.03	1.79	0.032
History of cancer (no)	448401	542	Reference							
History of cancer (yes)	3496	4	0.95	0.36	2.55	0.922	0.48	0.18	1.30	0.151
Smoking status										
Never smoker	92542	69	Reference							
Current smoker	229812	286	1.63	1.25	2.12	<0.001	1.90	1.46	2.48	<0.001
Former smoker	110832	156	1.81	1.36	2.41	<0.001	1.38	1.04	1.85	0.027
Missing	18711	35	2.59	1.72	3.89	<0.001	1.74	1.15	2.65	0.009
Alcohol intake										
Non-habitual drinker	77317	121	Reference							
Habitual drinker	332354	329	0.64	0.52	0.78	<0.001	0.76	0.62	0.94	0.012
Former habitual drinker	24075	63	1.60	1.18	2.17	0.003	1.08	0.79	1.47	0.625
Missing	18151	33	1.16	0.78	1.71	0.465	0.94	0.63	1.41	0.772
Walking hours per day										
< 0.5	210822	207	Reference							
≥ 1.0	81619	106	0.69	0.52	0.92	0.012	0.63	0.47	0.84	0.002
0.6-0.9	75883	103	0.92	0.67	1.26	0.591	0.79	0.58	1.08	0.142
0.5	43883	62	0.94	0.69	1.29	0.716	0.85	0.62	1.16	0.305
Missing	39691	68	0.95	0.59	1.51	0.816	0.59	0.35	0.98	0.040
Recruit (population-based versus)	172445	208	Reference							
Recruit (health examinees and/or volunteers)	279452	338	1.37	0.88	2.14	0.165	1.26	0.90	1.77	0.182
Regional random variance (SE)			0.18*	(0.974)			0.090	(0.258)		
-2Loglikelihood			6216*				5630			

* Regional random variance and -2loglikelihood of the univariate model were derived from the univariate model of area-level interest in screening.
