# Supplementary material for: A prospective cohort study of neighborhood stress and ischemic heart disease in Japan: a multilevel analysis using the JACC study data
Source: BMC Public Health. 2011 May 27;11:398. doi: 10.1186/1471-2458-11-398 (PMC3128019; doi:10.1186/1471-2458-11-398)
Supplement: Additional file 3 — Table 3: Mortality rate ratios (MRR) for ischemic heart disease in women [file 1471-2458-11-398-S3.PDF]

Table 3. Mortality rate ratios (MRR) for ischemic heart disease in women.

			Univariable				Multivariable			
	Person-year	No. of death	MRR	95%CI		p	MRR	95%CI		p
Area-level stress (per 1 percentage point)			1.09	0.99	1.20	0.066	1.07	1.00	1.14	0.057
Age (per 1 year)	664997	390	1.16	1.15	1.18	<0.001	1.16	1.14	1.17	<0.001
Stress										
High	73713	34	0.73	0.51	1.05	0.088	1.08	0.75	1.56	0.677
Moderate	60455	24	0.65	0.43	0.99	0.044	0.96	0.63	1.46	0.837
Low	416554	251	Reference				Reference			
None	114275	81	1.13	0.88	1.46	0.341	0.94	0.72	1.21	0.611
History of stroke (no)	659996	378	Reference							
History of stroke (yes)	5001	12	4.02	2.26	7.16	<0.001	1.68	0.94	3.01	0.082
History of hypertension (no)	531295	228	Reference							
History of hypertension (yes)	133702	162	2.77	2.26	3.40	<0.001	1.49	1.21	1.83	<0.001
History of ischemic heart disease (no)	650453	364	Reference							
History of ischemic heart disease (yes)	14544	26	3.40	2.24	5.16	<0.001	1.57	1.04	2.38	0.033
History of diabetes (no)	643322	339	Reference							
History of diabetes (yes)	21675	51	4.42	3.28	5.95	<0.001	2.57	1.90	3.47	<0.001
History of cancer (no)	9328	378	Reference							
History of cancer (yes)	655669	12	2.29	1.28	4.09	0.005	1.70	0.95	3.05	0.072
Smoking status										
Never smoker	30083	40	Reference							
Current smoker	8118	6	2.36	1.69	3.29	<0.001	2.68	1.91	3.76	<0.001
Former smoker	553862	286	1.34	0.60	3.01	0.480	0.93	0.41	2.11	0.868
Missing	72934	58	1.37	1.02	1.85	0.038	1.13	0.80	1.59	0.499
Alcohol intake										
Non-habitual drinker	145575	61	Reference							
Habitual drinker	10743	5	0.68	0.52	0.90	0.007	0.98	0.74	1.31	0.915
Former habitual drinker	463048	286	0.70	0.29	1.70	0.435	0.50	0.20	1.23	0.130
Missing	45631	38	1.11	0.78	1.57	0.552	0.89	0.60	1.34	0.590
Walking hours per day										
< 0.5	313851	169	Reference							
≥ 1.0	124501	76	0.73	0.52	1.03	0.075	0.78	0.55	1.10	0.160
0.6-0.9	101266	50	0.83	0.56	1.21	0.332	0.80	0.55	1.18	0.268
0.5	53623	40	0.65	0.43	0.98	0.042	0.61	0.40	0.92	0.019
Missing	71756	55	0.88	0.52	1.47	0.613	0.63	0.37	1.09	0.098
Recruit (population-based versus)	323441	152	Reference							
Recruit (health examinees and/or volunteers)	341556	238	2.27	1.29	3.99	0.004	1.43	0.95	2.16	0.087
Regional random variance (SE)			0.44*	(1.010)			0.140	(0.369)		
-2Loglikelihood			4854*				4105			

* Regional random variance and -2loglikelihood of the univariate model were derived from the univariate model of area-level interest in screening.
